# refineR: A Novel Algorithm for Reference Interval Estimation from Real-World Data

**DOI:** 10.1038/s41598-021-95301-2

**Published:** 2021-08-06

**Authors:** Tatjana Ammer, André Schützenmeister, Hans-Ulrich Prokosch, Manfred Rauh, Christopher M. Rank, Jakob Zierk

**Affiliations:** 1 Chair of Medical Informatics, Friedrich-Alexander-University Erlangen-Nuremberg, Erlangen, Germany; 2 Roche Diagnostics GmbH, Penzberg, Germany; 3 Department of Pediatrics and Adolescent Medicine, University Hospital Erlangen, Erlangen, Germany; 4 Center of Medical Information and Communication Technology, University Hospital Erlangen, Erlangen, Germany

**Keywords:** Diagnostic markers, Laboratory techniques and procedures

## Abstract

Reference intervals are essential for the interpretation of laboratory test results in medicine. We propose a novel indirect approach to estimate reference intervals from real-world data as an alternative to direct methods, which require samples from healthy individuals. The presented *refineR* algorithm separates the non-pathological distribution from the pathological distribution of observed test results using an inverse approach and identifies the model that best explains the non-pathological distribution. To evaluate its performance, we simulated test results from six common laboratory analytes with a varying location and fraction of pathological test results. Estimated reference intervals were compared to the ground truth, an alternative indirect method (*kosmic*), and the direct method (N = 120 and N = 400 samples). Overall, *refineR* achieved the lowest mean percentage error of all methods (2.77%). Analyzing the amount of reference intervals within ± 1 total error deviation from the ground truth, *refineR* (82.5%) was inferior to the direct method with N = 400 samples (90.1%), but outperformed *kosmic* (70.8%) and the direct method with N = 120 (67.4%). Additionally, reference intervals estimated from pediatric data were comparable to published direct method studies. In conclusion, the *refineR* algorithm enables precise estimation of reference intervals from real-world data and represents a viable complement to the direct method.

## Introduction

Clinicians rely on predetermined reference intervals for laboratory tests in order to properly interpret their patients’ test results^1,2^. Typically, reference intervals are determined using test results from a cohort of healthy reference individuals chosen from the population. The bounds of the reference interval are the values corresponding to the 2.5th and 97.5th percentiles of the distribution of test results^3^. The reference cohort must consist of a ‘sufficient’ number (≥ 120) of carefully selected and apparently healthy individuals who meet specific inclusion and exclusion criteria^3,4^. This way of sampling from a healthy cohort and establishing reference intervals from their test results is referred to as the ‘direct’ or ‘conventional’ method^3^. However, sample collection using this direct approach has many disadvantages. First, the definition of the term ‘healthy’ is ambiguous in clinical practice^4,5^. Sample collection is also time-consuming, costly, and represents an ethical challenge, especially in pediatrics^6–8^. Additionally, small sample sizes lead to sub-optimal precision of the estimated reference intervals^4,5^. Moreover, population-specific and (pre-)analytical differences, such as age, sex, ethnicity, and region lead to the need for population-based and laboratory-specific reference intervals. Such subgroup-specific reference intervals are very difficult to obtain due to the large effort in study planning and recruitment^4^. Thus, to further facilitate personalized healthcare, there is a clinical need for an alternative method.

So-called ‘indirect’ methods use existing data from routine measurements, often referred to as real-world data (RWD). RWD is generated continuously during patient care and check-up examinations, and includes non-pathological (physiological) and pathological test results. Under the assumption that the majority of test results are non-pathological, RWD can be employed to derive reference intervals using various statistical methods^3,4,9^. One advantage of the indirect methods is that the difficult task of defining ‘healthy’ individuals is not required. The real-world cohort contributing routine measurements for reference interval derivation closely matches the ‘intended-to-test’ population. In contrast, the direct method approach utilizes ‘super-healthy’ individuals, such as blood donors, to define reference intervals^9^. The indirect method also poses a faster and less expensive approach than the direct method, with fewer ethical concerns, especially in the pediatric field^6–8,10,11^. Additionally, due to the large number of measurements available, indirect methods can be expected to provide a substantial gain in precision of the estimated reference intervals. Furthermore, indirect methods can be employed to estimate reference intervals for developing countries. So far, population-specific reference intervals in developing countries are rare due to the substantial resources required for direct methods. Often reference intervals established in North America or Western Europe are inappropriately applied to local populations^12–14^. However, while indirect approaches alleviate challenges in data collection, they increase the challenge of data analysis.

Several indirect methods have already been implemented, e.g. the Hoffmann approach^15^ and the Bhattacharya method^16^. However, both are limited to a Gaussian distribution of non-pathological results, which is not applicable for most cases. Additionally, both methods require visual inspection, which leads to non-objective results and makes automation impossible. More recent methods, like the *RLE*^9,17–20^, the *TMC*^21^ or the *kosmic*^22^ algorithm assume that the non-pathological data can be modeled using a Box–Cox transformed normal distribution and can thus accommodate skewed, non-Gaussian distributions as well. The *RLE* (Reference Limit Estimator) is published as a freely available software on the website of the German Society for Clinical Chemistry and Laboratory Medicine (https://www.dgkl.de/verbandsarbeit/sektionen/entscheidungsgrenzen-richtwerte/), and optimizes the Kolmogorov–Smirnov distance between the cumulative densities of the model and the data^9^. The *TMC* (Truncated Minimum chi-square) operates on interval data and minimizes the chi-square (χ^2^) distance between the estimated and the observed counts within a truncation interval^21^. However, both methods are implemented using Microsoft Excel and the R software environment, which leads to a sub-optimal usability in practice. The most recently published indirect method, *kosmic*, which is an advancement of the *RLE* algorithm, is available as a command line application, python binding and web-based application as part of the PEDREF study (Next-Generation Pediatric Reference Intervals, www.pedref.org)^22^. Although *kosmic* already achieves good results and has addressed the practical limitations of the *RLE*, its performance decreases for datasets with a large fraction (> 20%) of pathological samples. Furthermore, for some datasets with unfavorable characteristics, computation time can be negatively impacted^22^.

In this work, we propose the *refineR* algorithm for the estimation of reference intervals. The novelty of this newly developed algorithm is that it pursues an inverse modeling approach to improve the quality of reference interval estimation in contrast to the forward approach used by other indirect algorithms. In addition, our algorithm provides a reasonable computation time and facilitates an unbiased application and simple operation, as no additional input parameters except the input data must be specified. The algorithm is available as an open-source R-package on CRAN [https://CRAN.R-project.org/package=refineR]. To evaluate the performance of the algorithm, we used simulated datasets based on routine data as well as patient samples (RWD) and compared *refineR* to the publicly available, peer-reviewed, and most recently published indirect method, the *kosmic* algorithm^22^, and the direct approach.

## Methods

Our algorithm for the estimation of reference intervals from RWD is based on the assumption that the majority of routine laboratory data is made up of non-pathological test results. Additionally, it is assumed that the distribution of these non-pathological samples can be modeled with a Box–Cox transformed normal distribution^23^, meaning a distribution that can accommodate normal as well as skewed distributions. Furthermore, the algorithm presumes that an interval of test results exists where the proportion of pathological test results is negligible. However, no assumptions are made about the location and distribution type of pathological samples in the joint distribution.

The *refineR* algorithm utilizes an inverse modelling approach. Here, the algorithm tries to find a model that can best explain the observed data in the original domain where the reference intervals are specified later on. In contrast, other published algorithms use a forward modelling approach by first transforming the data, then fitting a model to the data in the transformed domain. However, a resulting model in the transformed domain is not necessarily optimal in the original domain, meaning a small error in the transformed domain can result in a large error in the original domain. By utilizing the inverse approach, we circumvent this problem.

**Figure 1:**
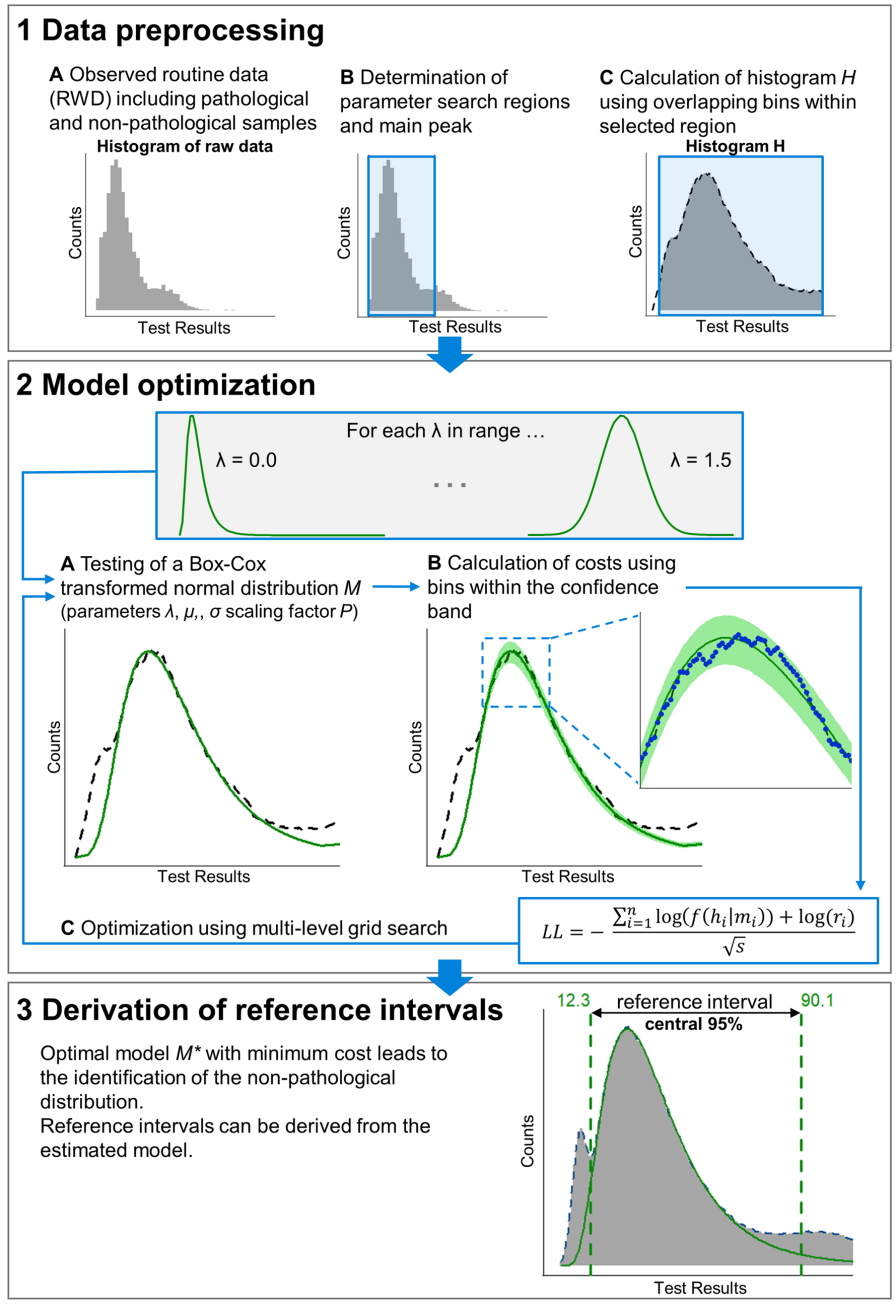
Flowchart of the *refineR* algorithm for the estimation of reference intervals from routine data. Based on the density of the observed routine data (**1A**), the search regions for the parameters *λ*, *μ* and *σ* are derived. This information is employed to define a region around the main peak (**1B**) where a histogram *H* of the data is calculated (**1C**, black dashed line). After that, a parametrical function *M* is employed to predict the expected values for each bin in *H* normalized with a factor *P* (estimated fraction of non-pathological samples) (**2A**, green line). An asymmetric confidence band around the expected values *M* (**2B**, green area) is utilized to identify the bins that then contribute to the calculation of the cost function (*LL*) (**2B** blue points). Using an optimization process (**2C**), the parameters λ*, μ*, σ*, and P* resulting in the minimum costs are identified and used to estimate the distribution of non-pathological samples. The corresponding reference intervals are then derived using this estimated distribution (**3**).

The main steps of the *refineR* algorithm for the estimation of reference intervals are as follows (a more detailed description is given in Fig. 1 and in the sub-sections).

1. Based on the density of the observed routine data, the parameter search regions are determined for the power parameter *λ*, mean *μ,* and standard deviation *σ*, defining the Box–Cox transformed normal distribution. Further, this information is utilized to define a region of test results that well characterizes the main peak. Within this selected region, a histogram representation *H* of the data is calculated.
2. Model Optimization by:
  2.A. Testing of a parametrical function *M*, a Box–Cox transformed normal distribution with the parameters (*λ*, *μ, σ*), to predict the expected values for each bin in the histogram *H*, normalized with a factor *P* describing the estimated fraction of non-pathological samples;
  2.B. Utilizing an asymmetric confidence band around the expected values *M* to identify the bins that most likely describe the non-pathological samples. These bins then contribute to the calculation of the cost function, which is based on the maximum likelihood approach. Assuming a Poisson distribution for the data in each histogram bin in *H*, it describes the likelihood that the observed data can be explained by the estimated model. (In order to minimize the cost function, we calculate the negative log-likelihood.)
  2.C. Using a multi-level grid search, the steps of testing a parameter set and evaluating the cost function are repeated to identify the parameters (λ*, μ*, σ*) and P* resulting in the minimum costs.
3. Identification of the non-pathological distribution using the optimized model M*, meaning the model comprised of the parameter set with minimum costs. The reference intervals are then derived from this estimated non-pathological distribution.

### Data preprocessing (determination of parameter search regions and main peak)

Within the first step, the algorithm preprocesses the data to find a region of test results that well characterizes the main peak and that ensures the exclusion of invalid values and values that do not contribute decisively to the main distribution (Fig. 1 1B). The main peak is defined as the one with the largest area under the curve (AUC), but not necessarily the one with the largest density or counts. Next, reasonable search regions for the parameters of the Box–Cox transformed normal distribution (*λ*, *μ* and *σ*) are derived. The parameter *λ* determines the skewness of the distribution with *λ* < 1 describing right-skewed distributions (positive skewness). A first special cases is at *λ* = 0, where the transformation results in a log-normal distribution. A second special case is obtained at *λ* = 1. Here, the Box-Cox transformation describes a Gaussian (normal) distribution. Values for *λ* > 1 yield left-skewed distributions (negative skewness). We use a fixed search region for *λ* between 0 and 1.5. This region covers the majority of observed distribution types in laboratory medicine following either a log-normal, right-skewed or a normal distribution. To account for rare cases where the distribution is slightly left-skewed, the range is extended to 1.5^24–26^. For values close to zero the effect of transformation is greater than for values around one, thus, we use a more fine-grained step size at 0: $\lambda ={x}^{1.54542}$ with $x\in \left[0.0, 1.3\right]$ by 0.1.

To define the search regions for *μ* and *σ,* and to find the region of interest, a Box–Cox transformation is applied to the input dataset for each respective *λ*. In the transformed space, the density of the data is estimated using the ash algorithm^27^ and the peak with the largest AUC is selected, following the assumption of the algorithm that the majority of the data is non-pathological.

At the selected main peak, the search region for *μ* is determined according to the following steps:

- Determine the width of the distribution at different heights (e.g. 50%, 55%, …, 95%) of the selected main peak
- Determine the center points of these widths as estimations for *μ*
- Calculate the range of these center points to define the search region for *μ*

At the selected main peak, the search region for *σ* is determined according to the following steps:

- Determine the width of the distribution at different heights (e.g. 50%, 55%, …, 95%) of the selected main peak
- Transform the peak width into standard deviations assuming a Gaussian distribution as the data has already been Box–Cox transformed
- Calculate the range of standard deviations to define the search region for *σ*

For a parameter *λ* close to its optimal value, we expect a symmetrical, Gaussian-like density distribution in the transformed space. For such symmetrical distributions, the distances on both sides of the main peak are equal, and thus the estimated ranges for *μ* and *σ* are expected to be minimal, when normalized by their mean values. Utilizing this assumption, the following steps determine the region of test results that is of interest:

- Select *λ* where $\sqrt{{\text{normalized range}(\mu )}^{2}+ {\text{normalized range}(\sigma )}^{2}}$ is minimal
- Calculate the region of test results in transformed space using the respective *μ* and *σ* of the selected *λ*: $\text{mean}\left(\mu \right)\pm 3\times \text{mean}(\sigma )$
- Apply the inverse Box–Cox transformation to translate the calculated region to the original space.

After defining the region of interest based on the estimated parameter search regions, we generate a histogram representation of the data within this region by sorting the data into a histogram with overlapping bins (Fig. 1 1C). Utilizing overlapping bins is a compromise between ensuring a minimum amount of noise in each bin and achieving a smooth representation of the distribution of the data.

### Optimization process

After data preprocessing, the *refineR* algorithm tries to find the optimal model describing the underlying data by utilizing a multi-level grid search for the optimal values λ*, μ* and σ* (Fig. 1 2C). Furthermore, a scaling factor *P*, the fraction of non-pathological samples, is optimized within a defined search region for each combination of *λ, μ* and *σ*. In a first step of the optimization, the algorithm calculates the costs for each test combination (*λ, μ* and *σ*) and selects the combination, where the costs are at their minimum. In a next step, the search region around these preselected values is explored on a denser grid.

### Estimation of a parametrical model

Within the optimization process, the density of a Box-Cox transformed normal distribution *M* is calculated for each parameter combination *λ, μ* and *σ* (Fig. 1 2A). Initially, the height of this model and the height of the actual histogram bins differ. Thus, we scale the model with the factor *P *(*P* ≤ 1). To find the optimal *P*, we first compute the ratio of the observed counts and the model at different regions adjacent to the peak, the peak itself, as well as confidence intervals for each of these estimations. We then use the minimum of both confidence limits to define the search region in which *P* is varied. Due to the fact, that our estimated model describes the expected value for each bin, we can define a confidence band around the estimation based on Poisson statistics. In our case, this region is constructed in such a way that it covers the observed counts from the non-pathological distribution in 99% of the cases, when the underlying model is correct (Fig. 1 2B). As pathological samples within the data only add a positive bias to the total histogram counts, we try to counteract this potential bias by using an asymmetric confidence region. The higher the fraction of pathological samples, meaning higher positive bias is expected, the stronger the asymmetry. As a consequence, an overestimation of reference intervals (meaning too broad ranges) can be reduced.

### Calculation of costs

After defining the confidence band, the algorithm selects the bins that fall within this region of the model (Fig. 1 2B). The selected bins then contribute to the calculation of costs utilizing a negative log-likelihood function with regularization (Eq. 1). The term $f({h}_{i}|{m}_{i})$ describes the likelihood of observing counts in bin *h*_*i*_ given the expected histogram counts in this bin, which are Poisson distributed with *m*_*i*_*.* The regularization term *r*_*i*_ describes the number of counts in the respective bin *i*, therefore rewards bins with higher counts (as these most likely describe non-pathological samples) and penalizes bins with lower counts (as these are more likely to originate from pathological samples). The parameter ($\sqrt{s}$) in the denominator represents the number of chosen bins. In the cost function, it thus penalizes cases where (too) many bins are selected.

1 $$LL=- \frac{\sum_{i=1}^{n}\mathit{log}\left(f\left({h}_{i}\right|{m}_{i}\right))+log({r}_{i})}{\sqrt{s}}$$

### Calculation of confidence intervals using bootstrapping

To provide confidence intervals for the estimation of reference intervals, we use bootstrapping^28^. Here, we randomly sample the original dataset *n*-times with replacement, with *n* being the size of the dataset. This randomly drawn set then serves as input for the *refineR* algorithm. The steps of randomly drawing samples and estimation of the model are repeated a certain number of times (e.g. 200). The 95% confidence interval is then computed as the central 95% region of the 200 estimated lower and upper reference limits, respectively.

### Description of evaluation data and methods

**Figure 2:**
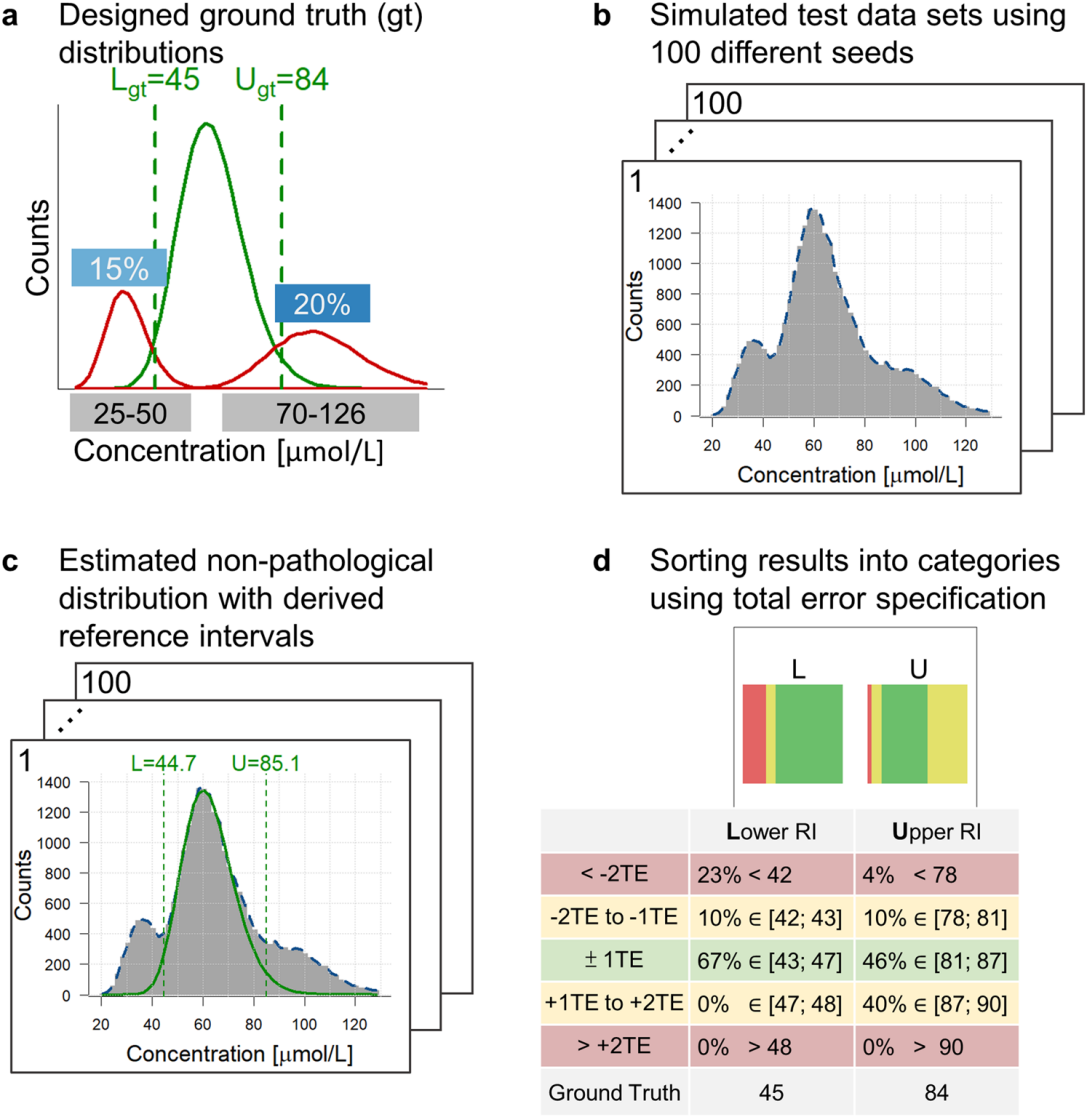
Description of generating simulated test cases and benchmarking the resulting estimations using an example picked from Fig. 3b (‘CREA’). (**a**) Example of designed ground truth distribution for ‘CREA’ in green with added pathological distributions left (25–50) (15%) and right (70–126) (20%) of the non-pathological distribution in red. (**b**) Simulated test datasets using 100 different seeds that serve as input to the algorithms. (**c**) Results obtained using the *refineR* algorithm on datasets shown in (**b**) with the green horizontal dashed lines showing the estimated reference interval and the green curve showing the estimated non-pathological distribution. (**d**) Visualization of the results for the simulated datasets shown in (**b**) with 100 different seeds and applying the algorithm on the various datasets (**c**). The obtained estimated reference intervals are grouped into the color-coded categories regarding their deviation from the ground truth (Table 1). *L* lower reference limit, *U* upper reference limit, *TE* total error, *RI* reference interval.

To evaluate the performance of the *refineR* algorithm, we use simulated datasets, as this has the advantage that we know the underlying “ground truth” and thus can evaluate the performance of the algorithm in a quantitative way. We simulated data for the following six biomarkers: “Alkaline phosphatase, ALP”, “Creatinine, CREA”, “Hemoglobin, Hb”, “Free thyroxine, FT4”, “Thyroid-stimulating hormone, TSH”, “Gamma-glutamyltransferase, γ-GT”. The simulations were designed to mimic RWD and to identify the limitations of the algorithm. The distribution of ‘Hb’, ‘TSH’ and ‘γ-GT’ were adapted from Zierk et al.^22^, while the others were constructed independently based on real routine measurements. We analyzed the distributions of these biomarkers using a dataset of several thousand routine measurements. Then we designed the simulations to mimic non-pathological and pathological distributions in accordance with the appropriate reference interval. We generated random Box–Cox transformed normal distributions of non-pathological test results and added pathological samples with varying position and fraction. One example of a designed test case is shown in Fig. 2a. The datasets were then generated using 100 different seeds to minimize the influence of random effects (Fig. 2b).

We applied both the *refineR* and the *kosmic* algorithm^22^ to the same dataset (Fig. 2c) to compare the performance of our proposed algorithm to the publicly available, peer-reviewed and most recently published indirect method. For performance evaluation, besides reporting the upper and lower reference limit for each result, we calculated error and bias averaged over all test cases:

2 $$\text{Mean percentage error: } MPE= \frac{1}{n}\times \sum_{i=1}^{n}\frac{|r{i}_{i}-gt|}{gt},$$

3 $$Relative\,Bias=\frac{1}{n}\times \sum_{i=1}^{n}\frac{r{i}_{i}-gt}{gt},$$

where *gt* = ground truth for lower and upper reference limit, *ri*_*i*_ = reference interval *i*, and *n* = number of (simulated) cases.

For a convenient comparison of the quality of results, we also grouped the estimated reference intervals into five different categories, defined using the total error specification from the EFLM (European Federation of Clinical Chemistry and Laboratory Medicine) biological variation database^29^ (Fig. 2d). We decided to use the total error as a benchmark, as it considers both the biological variability and the analytical error for each marker. The total error specifications for the different markers are shown in Table 1.

**Table 1:**
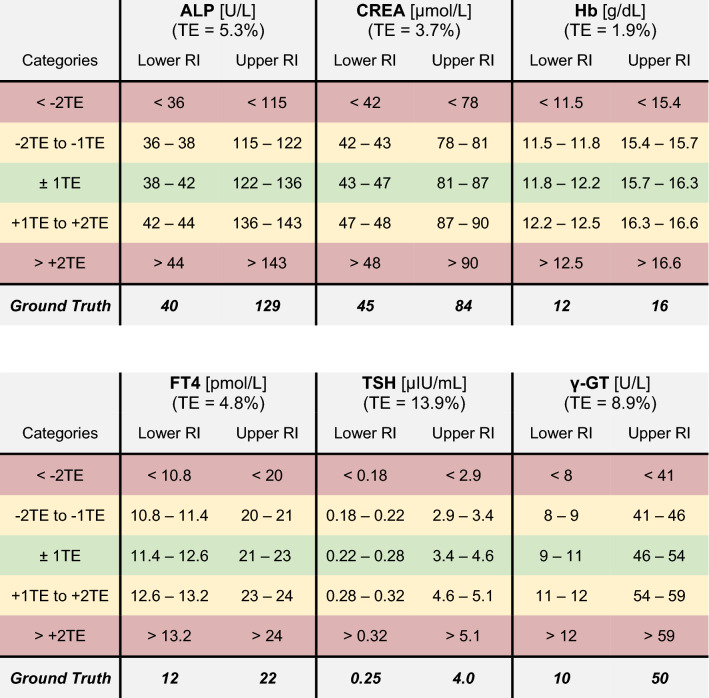
Definition of evaluation categories for different simulated analytes using the total error specification.

The table shows for each simulated biomarker the ranges of the absolute deviation from the ground truth used for defining the color-coded evaluation categories.

*TE* total error, *RI* reference interval.

Legend (adapted from Zierk et al.^22^): Green (Within ± 1TE)—Appropriate for RI estimation. Yellow (Within ± 2TE)—Appropriate for quality control. Red (Outside ± 2TE)—Inappropriate estimations. Use with caution in select settings.

Additionally, we conducted a comparison of the indirect methods to the direct method, which serves as a baseline benchmark, with N = 120 and N = 400 reference samples. Although the direct method is considered the ‘gold standard’, its results also include statistical uncertainty, due to sampling bias. We simulated this approach by drawing N = 120 and N = 400 random samples from the simulated non-pathological distribution for the various analytes. These data points were then used to determine the 2.5th and 97.5th percentile as reference intervals. To account for the uncertainty from random sampling, sampling was repeated 10,000 times.

Furthermore, we examined the performance of *refineR* in pediatric datasets obtained from the laboratory information system of a tertiary care center (Department of Pediatrics and Adolescent Medicine, University Hospital Erlangen, Germany). We retrieved patient samples for the six biomarkers we previously simulated (ALP, CREA, Hb, FT4, TSH, γ-GT) for patients aged 3–18 years. The different age groups per analyte are shown in Supplementary Table S7, together with the sex of the underlying cohort, the number of samples and their measuring unit. The subgroups were selected based on observed minor dynamic in the measured test results^6–8,30^. The measurements were performed on Roche cobas instruments (ALP, CREA, FT4, TSH, γ-GT) and SYSMEX instruments (HB). For these biomarkers, we estimated reference intervals using *refineR* and *kosmic* with N = 200 bootstrap iterations and compared the results to reference intervals obtained from different direct method studies from literature^31–40^. The comparison studies include reference intervals obtained as part of the CALIPER project (*CA*nadian *L*aboratory*I*nitiative in *PE*diatric *R*eference Intervals)^31–35^, package inserts accompanying the analytical devices^38–40^, a study obtained on a German pediatric cohort^37^ and one carried out in an Austrian adolescent cohort^36^. Sample sizes in comparison studies were between 26 and 1872 (median: 200). More detailed information can be found in Supplementary Table S8. Use of pseudonymized pediatric and adult patient datasets obtained during patient care without patients’ explicit consent is in accordance with the applicable German/Bavarian regulations and has been approved by the Ethical Review Boards of the University Hospital Erlangen, reference number 97_17 Bc.

The *refineR* algorithm was evaluated on a standard notebook with dual core CPU and 16 GB of memory using the R version 3.5.1^41^. The algorithm is available as an open-source package on CRAN [https://CRAN.R-project.org/package=refineR]. The following results were calculated with the *refineR* package version 1.0.

## Results

We provide a high-performance and robust open-source implementation of an indirect method to accurately estimate reference intervals using RWD, which is available as an R-package on CRAN.

**Figure 3:**
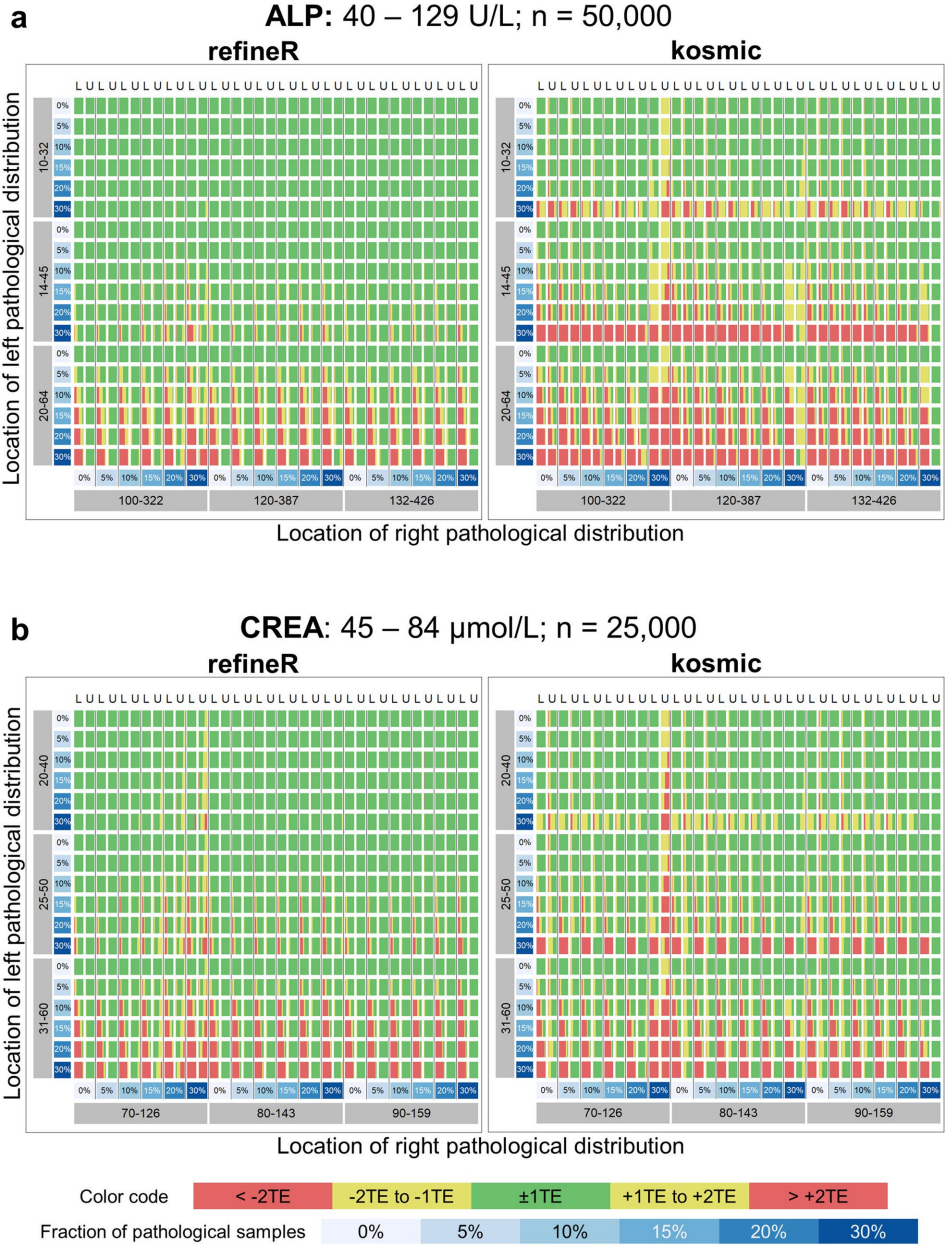

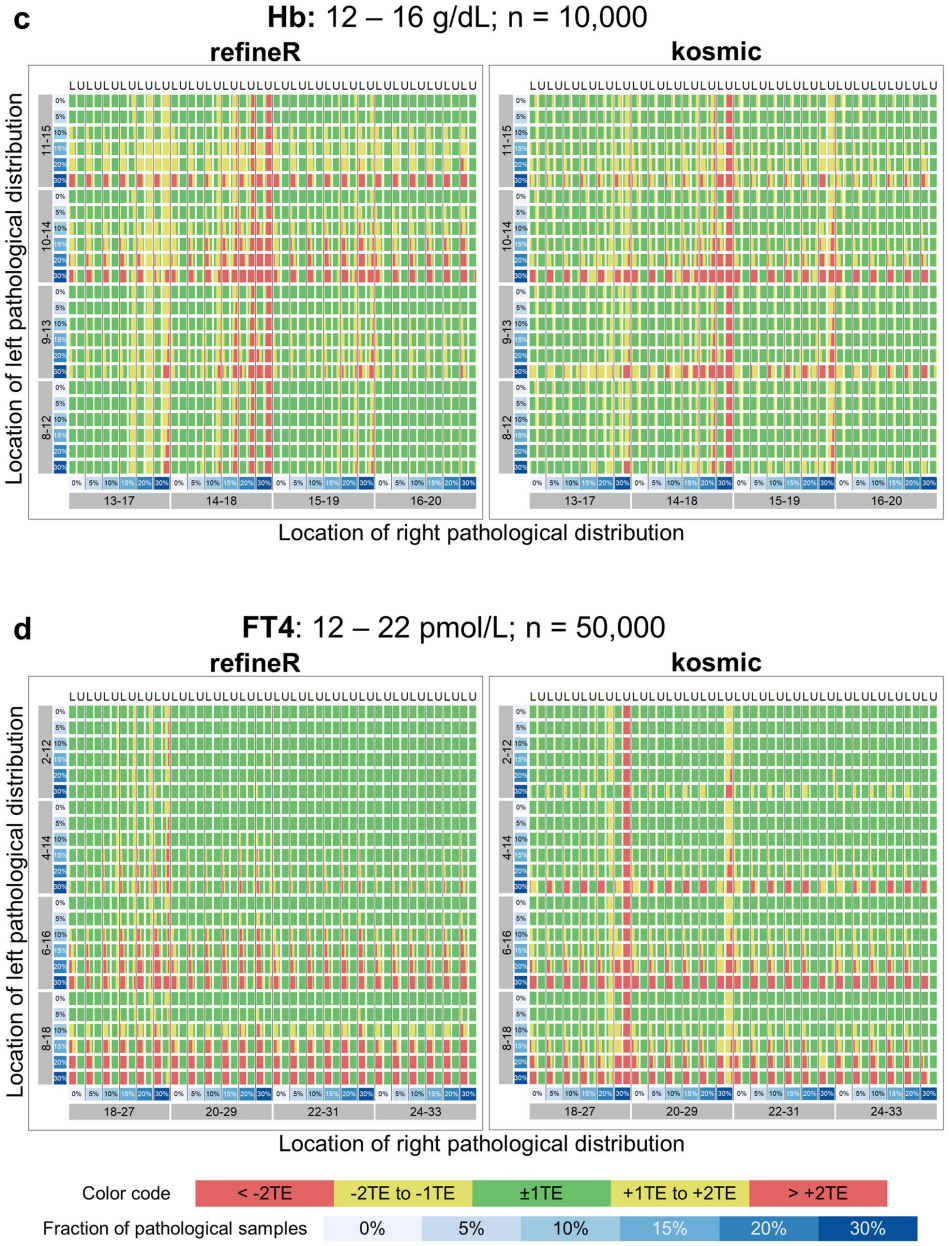

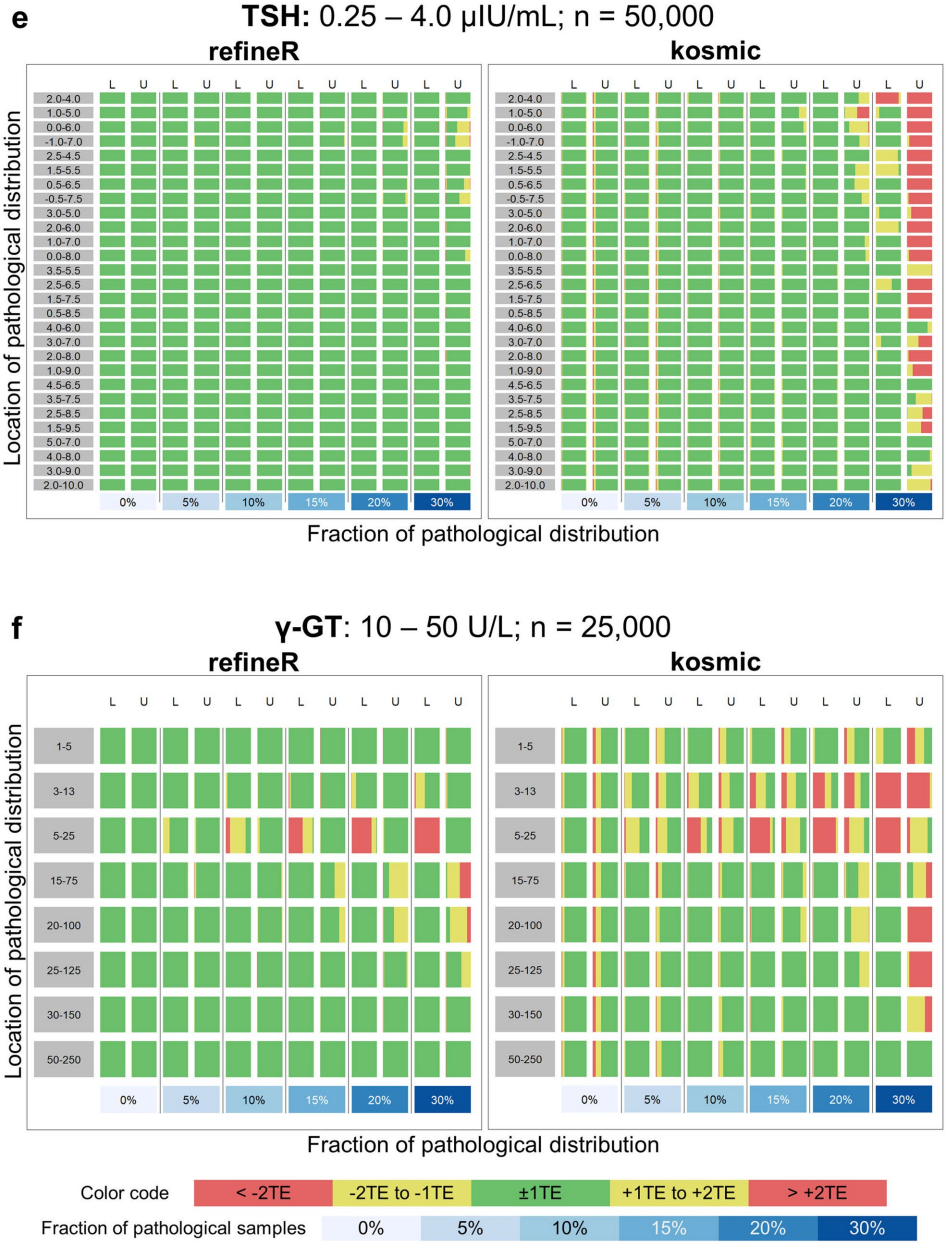
Comparison of *refineR* and *kosmic* algorithm for the simulated analyte distributions. Each plot shows the distribution of the estimated reference intervals in presence of abnormally low and high values in the dataset. (**a**–**d**) The rows represent the pathological distribution added on the left side, and the columns the pathological distribution added on the right side of the non-pathological one. The blue-shaded boxes indicate the different pathological fractions. Two adjacent columns always correspond to the same distribution indicating the results for the lower (L) and upper (U) reference limit. Each color-coded box in the matrix shows the distribution of the results obtained from 100 different seeds within the five evaluation categories (see “Methods” Table 1, Fig. 2) for a specific combination of pathological fraction and location. (**e**–**f**) Each row shows the position of the pathological distribution and each column shows the fraction of pathological samples of the whole dataset from 0 to 30%. *TE* total error.

To investigate the performance of our proposed *refineR* algorithm, we used simulated test cases for six different analytes with a varying fraction and location of the pathological distribution (Fig. 2). By adding different pathological distributions, we obtained test cases with varying levels of difficulty. The matrices in Fig. 3 show that the performance of the *refineR* algorithm depends on the location and the fraction of the pathological distribution(s). The associated percentages accompanying these distributions are provided in the Supplementary Tables S1–S6. For test cases with a minor overlap between the pathological and non-pathological distribution, *refineR* achieved results very close to the ground truth even for a high fraction (≥ 20%) of pathological samples (Fig. 3a,b upper right part of matrix for ‘ALP’, ‘CREA’, Fig. 3c,d lower right and upper right part for ‘Hb’ and ‘FT4’ respectively, Fig. 3e ‘TSH’, Fig. 3f ‘γ-GT’). Additionally, for the cases with a low pathological fraction (≤ 15%), the majority of estimated reference intervals were within ± 1 total error deviation from the ground truth (Fig. 3a–d where the sum of pathological distributions is ≤ 15%, Fig. 3e ‘TSH’, Fig. 3f ‘γ-GT’). For very challenging simulated datasets, such as test cases with a high overlap between the distributions, *refineR* showed an increasing deviation from the ground truth (Fig. 3d highly overlapping ‘FT4’ and Fig. 3c ‘Hb’ cases with high fraction of pathological ≥ 20%, Fig. 3f ‘γ-GT’ third row/fourth and fifth row with a pathological fraction ≥ 20%). For test cases with an extremely high pathological fraction (up to 60%), the estimations increasingly deviated from the true reference limits. (Note that for the performance evaluation, we violated the assumption underlying *refineR*, that the majority of samples are non-pathological and that a region of test results exists where the amount of pathological samples is negligible.) However, for cases with a high fraction but less overlap, we observed that *refineR* generally yielded reliable estimations (Fig. 3a,b 1st and 2nd ‘grey’ row and column, Fig. 3c ‘Hb’ 3rd and 4th ‘grey’ row and column and Fig. 3d ‘FT4’ 1st and 2nd ‘grey’ row and 2nd–4th column).

To compare the performance of the *refineR* algorithm to another indirect method, we applied the *kosmic* algorithm to the same simulated biomarker distributions. For *refineR*, 65.7–98.7% (overall mean of 82.5%) of the estimated reference intervals for all test cases were within one total error deviation from the ground truth (Table 2, Supplementary Tables S1–S6). For the established *kosmic* algorithm, 58.0–84.4% (overall mean of 70.8%) of estimates fulfilled that requirement showing that the proposed *refineR* algorithm yielded more results within one total error deviation. For the individual test cases (Supplementary Tables S1–S6), *refineR* also showed a higher amount within this category for all simulated analytes except for ‘Hb’. Here, *kosmic* obtained slightly better agreement with the ground truth than *refineR* (68.2% and 65.7% within ± 1 total error deviation, respectively, Supplementary Table S3). In correspondence to the analysis above, *refineR* revealed more results within one total error deviation from the ground truth than *kosmic* in five out of the six simulated analyte test sets and overall. A summary of the calculated mean percentage error and relative bias for the different test cases is shown in Table 3. These findings show, consistent with the other results, a smaller error for the *refineR* algorithm than for *kosmic* in five out of the six simulated biomarker distributions as well as a smaller bias in all cases. Overall, *refineR* obtained a mean percentage error of 2.77% while *kosmic* ended up with 5.78% (Table 3).

**Table 2:**
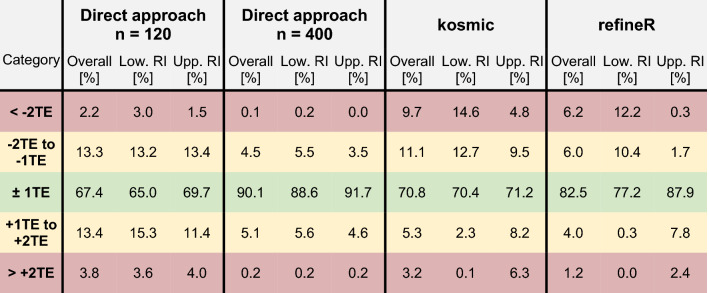
Summary table with comparison of quality of results for all analyzed test cases.

The table shows for all considered methods (direct method with n = 120 and n = 400, *kosmic* and *refineR* algorithm) the overall distribution of RI estimates for the six simulated analyte distributions in the five evaluation categories (see “Methods” Table 1). The results are shown for the lower and upper reference interval as well as an average of both options for the overall amount.

*TE* total error, *RI* reference interval.

To investigate how the *refineR* algorithm compares to the direct approach, we simulated the direct method using 120 and 400 simulated “apparently healthy” individuals and grouped the results in the color-coded categories, as shown in Tables 2 and 3 (and Supplementary Tables S1–S6). For the direct approach using 120 samples, *refineR* showed a higher amount within one total error deviation from the ground truth for the (highly) skewed distributions, like ‘TSH’ or ‘ALP’ (Supplementary Tables S1, S2, S5, S6). For Gaussian-like non-pathological distribution, the direct approach with 120 data points obtained more estimates within this category than the *refineR* algorithm (Supplementary Table S3 (‘Hb’), Table S4 (‘FT4’)). Overall, this direct method yielded a range of 55.0–81.9% (mean of 67.4%) of estimates within one total error deviation from the ground truth. These findings are also represented in the error, with a smaller error for the cases ‘Hb’ and ‘FT4’ and a larger error for the other test cases compared to *refineR*. Looking at the results for the direct approach with 400 samples, we again observed that for the highly skewed distributions like ‘TSH’, *refineR* reached 98.7% within the category defined as appropriate for reference interval estimation and the direct approach reached 85% within this range (Supplementary Table S5). For the other less skewed simulated biomarker distributions, the direct approach with 400 samples achieved a higher proportion within one total error deviation from the ground truth compared to *refineR*. Altogether, the method obtained 81.7–97.7% (mean of 90.1%) within this category. The overall calculated error for this direct method was 3.14%, indicating that *refineR* yielded the smallest error of all analyzed methods. The bias however was smallest for the direct approach utilizing 400 samples (Table 3).

**Table 3:** Mean percentage error and relative bias for each simulated analyte and overall.

Marker	Performance measure [%]	Direct approach n = 120	Direct approach n = 400	kosmic	refineR
ALP	MPE	5.51	**3.11**	*8.15*	3.33
	Rel. Bias	0.24	**0.04**	*− 6.79*	− 2.37
CREA	MPE	2.93	**1.66**	*3.83*	2.63
	Rel. Bias	0.05	**0.00**	*− 2.84*	− 1.43
Hb	MPE	1.37	**0.78**	1.60	*1.67*
	Rel. Bias	0.00	**− 0.01**	*0.18*	0.06
FT4	MPE	2.88	**1.63**	*3.83*	3.51
	Rel. Bias	0.04	**0.00**	− 1.32	*− 2.18*
TSH	MPE	*13.17*	7.37	8.39	**2.12**
	Rel. Bias	1.5	**0.36**	*1.55*	0.41
γ-GT	MPE	7.58	4.27	*8.90*	**3.37**
	Rel. Bias	0.48	**0.10**	*− 5.91*	− 0.61
Overall	MPE	5.57	3.14	*5.78*	**2.77**
	Rel. Bias	0.39	**0.08**	− 2.52	− 1.02

The table shows for each analyzed test case the calculated performance measurements for all considered methods (direct method with n = 120 and n = 400, *kosmic* and *refineR* algorithm). The performance for each individual test case as well as the mean performance over all six test cases are shown.Values in bold represent the best result per biomarker (smallest MPE/Rel. Bias closest to zero), while the values in italics represent the worst result (largest MPE/Rel. Bias farthest from zero).

*MPE* mean percentage error.

**Figure 4:**
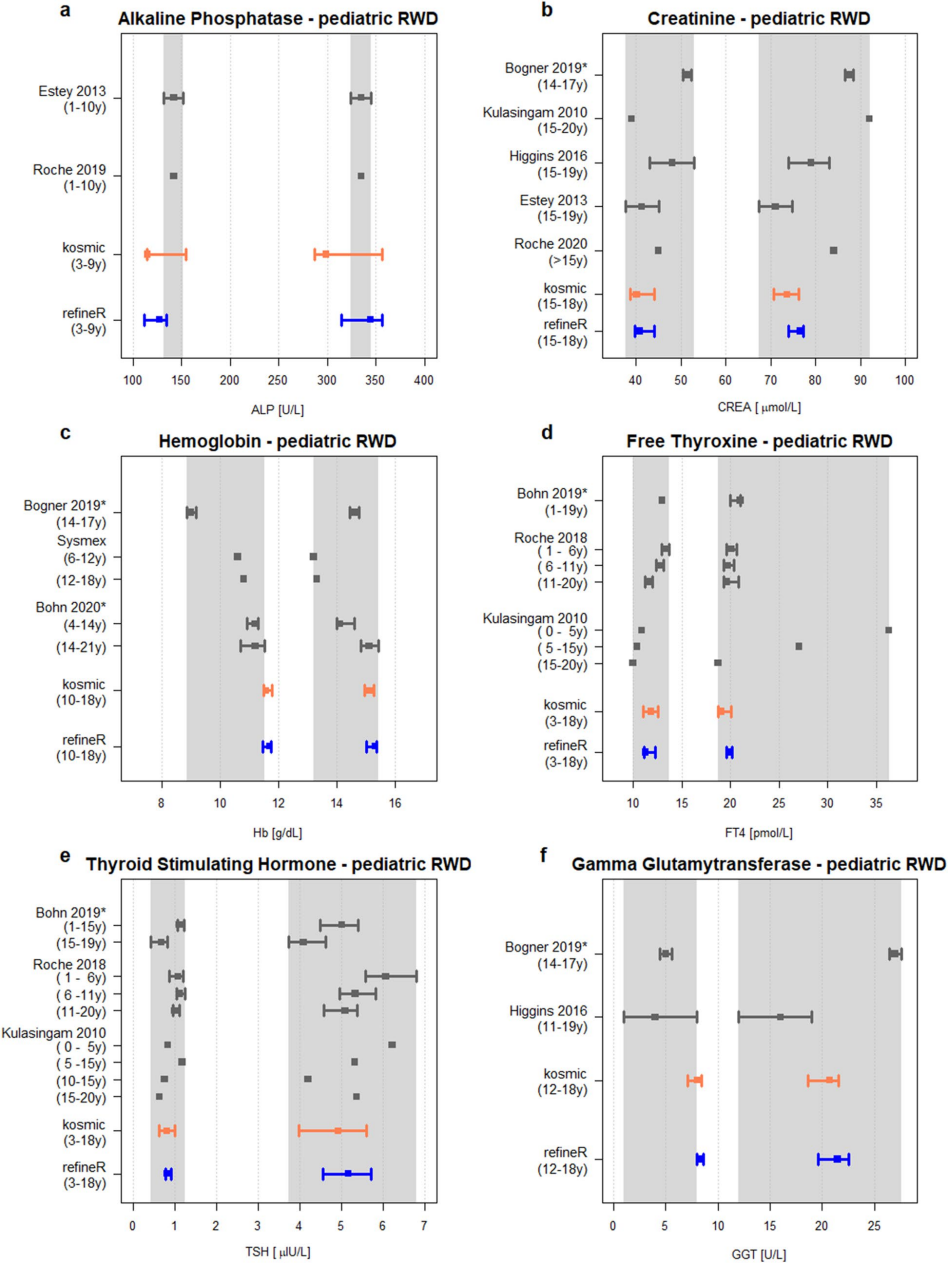
Comparison of reference intervals estimated with the *refineR* and *kosmic* algorithm using pediatric RWD to reference intervals published in literature. Each plot (**a**–**f**) shows for the indicated biomarker the estimated lower (left) and upper (right) reference limit obtained using *refineR* (blue) and *kosmic* (orange) in comparison to published reference intervals established using direct methods (grey)^31–40^. The squares represent the point estimates while the whiskers show the 95% confidence interval (* indicates 90% confidence interval). The lightgrey area illustrates the margin of variation of the different direct studies (meaning the range from the minimum lower confidence limit (or point estimate) to the maximum upper confidence limit (or point estimate)). Please note that age ranges between indirect methods and published direct methods do not perfectly match. The numeric values for the reference intervals can be found in Supplementary Table S8.

To evaluate how the *refineR* algorithm performs on RWD we applied both indirect methods, *refineR* and *kosmic* to patient data obtained from the Department of Pediatrics and Adolescent Medicine, University Hospital Erlangen. The results were compared to published reference intervals established with direct methods (Fig. 4 and Supplementary Table S8).

The reference intervals estimated with the two indirect methods, *refineR* and *kosmic*, showed good agreement for all six analyzed biomarkers, indicated by their highly overlapping confidence intervals. Analysis of reference intervals from direct method studies published in literature shows that different studies yielded heterogeneous results, indicated by the lightgrey colored margin of variation (e.g. Fig. 4b CREA, Fig. 4d FT4). Further, it can be observed that the age ranges reported in literature differ. To cover the age range of routine measurements employed for indirect methods, we included multiple age groups in the comparison. As can be seen in Fig. 4, the estimated confidence intervals of the indirect methods overlap for each biomarker with the margin of variation of the different direct studies (lightgrey area). For ALP, the indirect methods revealed a slightly wider reference interval than obtained from literature due to smaller values of the lower limit (Fig. 4a). In contrast, for Hb and γ-GT *refineR* and *kosmic* estimated a narrower reference interval due to higher values of the lower reference limit compared to the direct methods (Fig. 4c,f, Supplementary Table S8).

To compare computation time of the *refineR* algorithm to the established *kosmic* algorithm, we picked a representative subset of ten examples of each of the six simulated analyte distributions and computed the results with both algorithms on a standard notebook with dual core CPU and 16 GB of memory. The *refineR* algorithm required an overall computation time for all N = 60 datasets of about 2.7 min (mean 2.53 s. median 2.53 s, min 1.6 s, max 5.61 s) compared to *kosmic* which took about 124 min (mean 124 s, median 7.55 s, min 0.39 s, max 1070 s).

## Discussion

Clinical laboratories are strongly advised to determine their own reference intervals to account for population-specific and laboratory-specific (pre-)analytical biases^3^. However, using the direct approach with a sufficient amount (≥ 120) of apparently healthy individuals poses a major challenge for laboratories. Especially in pediatrics, ethical restrictions often limit sample size while the high prevalence of chronic disease and medication use in the elderly demonstrates the dilemma of selecting “healthy” or “super-healthy” individuals. Indirect methods may therefore offer an easier, more time-efficient and less costly way for individual laboratories to establish their own laboratory-specific reference intervals. Furthermore, using the estimated model of the non-pathological distribution obtained with the indirect method allows for the establishment of standardized laboratory values by computing z-scores (i.e. assessing their relative position within the distribution), thus simplifying the interpretation of test results as well as the comparison of different laboratories and manufacturers^30^.

With the *refineR* algorithm, we provide an efficient and robust implementation of an indirect method for the estimation of reference intervals. The algorithm can compute reference intervals in a time-efficient manner even for large datasets and thus enables the computation of confidence intervals using bootstrapping within a reasonable runtime. In contrast to *kosmic* where unfavorable data set characteristics can lead to long computation times, the runtime of *refineR* is not impacted by the features of the dataset^22^. Additionally, *refineR* enables an unbiased and straightforward application in practice, as it does not require the specification of any additional input parameters except the input dataset. The algorithm is provided as an open source R-package and can thus be easily integrated into custom analysis pipelines or laboratory information systems, thereby providing laboratories with an alternative to the direct method to establish their own reference intervals.

In this work, we have shown that the novel *refineR* algorithm achieves robust results for both less and more challenging test cases. Particularly for simulated distributions with a high (> 20%) proportion of pathological samples, *refineR* yielded results that were superior to *kosmic* (Fig. 3). We challenged the algorithms with test cases containing two pathological distributions each contributing up to 30% to the overall distribution, thereby violating the prerequisite for a substantial majority of non-pathological test results, which applies to all indirect methods. Nevertheless, *refineR* was shown to provide appropriate estimates for test cases when the overlap between the non-pathological and the pathological distributions was not too high, meaning there still exists a minor region, where only non-pathological samples are present. Furthermore, our results showed that the tolerance of pathological samples (overlap or abundance) was higher within the *refineR* algorithm than compared to *kosmic*. *refineR* still produced clinically usable reference intervals for difficult test cases, whereas reference intervals estimated using *kosmic* had an unacceptable bias (e.g. Fig. 3).

When comparing the performance of *refineR* to the direct method, *refineR* achieved better outcomes than the direct method with N = 120 for the majority of cases. For the approach using N = 400, the direct method outperformed *refineR* in most cases. For highly skewed distributions like ‘TSH’ or ‘γ-GT’ with only one pathological distribution present, *refineR* yielded reference interval estimations of higher accuracy. Although it is recommended to use at least N = 400 samples^42^, the official guideline describes the minimum sample size with N = 120^3^, which is commonly used in laboratory practice. Recruiting 400 healthy individuals is associated with substantial financial, logistical, and time constraints and thus is often not considered feasible in practice. The slightly worse results for *refineR* compared to *kosmic* for ‘Hb’ or compared to the direct method for ‘Hb’ and ‘FT4’ may originate from the great number of cases with a high overlap between the pathological and non-pathological samples as well as from the Gaussian-like shape of the non-pathological distribution that reduces the uncertainty of the direct sampling (in comparison to skewed distributions). The overall performance (Table 3) showed that *refineR* outperforms the other analyzed methods, the established indirect approach, *kosmic*, as well as the two direct approaches regarding the mean percentage error. For certain test cases (such as ‘ALP’ and ‘γ-GT’), the bias of the direct approach was close to zero despite having large mean percentage errors. This can be explained by the fact that the bias can have both positive and negative values that can cancel each other out. As a consequence, the mean bias does not assess the quality of the individual estimates. In contrast, the mean percentage error is calculated using an absolute difference, and thus can be used to evaluate the quality of the individual results. The analysis of the direct approach also shows that the direct method can have a high uncertainty itself that decreases with the number of samples and is influenced by the distribution of the biomarker of interest^3^.

When applying both indirect methods to RWD, we observed that both methods yielded similar results (overlapping confidence intervals for each analyte). Furthermore, the estimated results were comparable to the reference intervals obtained in different direct method studies, showing that both indirect methods are capable of estimating precise reference intervals in real-world scenarios.

Differences in the estimated reference intervals among the various direct method studies and between direct and indirect methods can best be explained by differences in the underlying population. First, for some biomarkers, age partitions do not agree perfectly between the different studies and our obtained dataset (e.g. Fig. 4c–e). Second, the geographic and ethnic composition of the analyzed cohorts varies. For example, we compare reference intervals obtained for children in Canada (CALIPER studies ^31–35^), the US^40^, Austria^36^, and Germany^37^. Third, the time range of measurement and measurement sites differ leading to potential time- and site-specific effects although we compared only studies using Roche cobas or Sysmex instruments. Furthermore, the rather small sample size of some direct method studies can bias the results. The observed variations emphasize the fact that the clinical truth of the reference intervals is unknown. In summary, these results show that indirect methods can be used to calculate reference intervals comparable or even superior to the direct method.

## Limitations

The presented evaluations of the *refineR* algorithm using simulations show that the method can be used to estimate reference intervals for homogenous populations of different sizes in a robust manner. However, many reference intervals are age-dependent or vary depending on other covariates, like sex or ethnicity. Thus, future work will focus on the enhancement of this one-dimensional approach to incorporate covariates. Specifically, the generation of continuous reference intervals depending on the age of the individual will be explored, as the establishment of such reference curves would provide important insights for children^6–8,43^ and the elderly^44^.

We have shown that the *refineR* algorithm outperforms *kosmic*, the publicly available, peer-reviewed, and most recently published indirect method, in the majority of test cases, by intensively studying the performance using six common and medically relevant biomarkers. Nevertheless, there is still the need for a standardized test database covering an even broader variety of medically relevant, simulated distributions of biomarkers, as well as a systematic evaluation of different methods.

While we have shown that the *refineR* algorithm already achieves promising results, the assumption that the non-pathological samples can be modeled with a Box–Cox transformed normal distribution may not be appropriate for all biological distributions^42^. Thus, in future work, we will examine if using other transformations like the modified Box–Cox transformation^45^ may improve *refineR’s* performance.

## Conclusion

We developed the *refineR* algorithm for the precise and time-efficient estimation of reference intervals using real-world data from laboratory information systems. *refineR* is provided as an open source (GPL v3) R-package on CRAN. Our results show that *refineR* outperforms both the direct method with a reference population of 120 individuals and the publicly available, peer-reviewed, and most recently published indirect method algorithm, *kosmic* using simulated data. For the patient datasets, the results are within the margin of variation of different direct method studies. By requiring less resources and facing fewer ethical issues, *refineR* is a viable alternative to the direct approach.

## Supplementary Information

Supplementary Tables.

Supplementary Information.

## Data Availability

An open-source (GPL v3) R-package is available at https://CRAN.R-project.org/package=refineR. The R code/scripts for generating the simulated evaluation datasets and reproducing the *refineR* results are included as supplementary material to this published article. The patient datasets analyzed in the present report (Fig. 4, Supplementary Tables S7 and S8) were used with permission from Prof. M. Rauh (Department of Pediatrics and Adolescent Medicine, University Hospital Erlangen, Erlangen, Germany) and are not publicly available. Data are however available from the authors upon reasonable request and with permission from Prof. M. Rauh.
