# Supplementary material for: refineR: A Novel Algorithm for Reference Interval Estimation from Real-World Data
Source: Sci Rep. 2021 Aug 6;11:16023. doi: 10.1038/s41598-021-95301-2 (PMC8346497; doi:10.1038/s41598-021-95301-2)
Supplement: Supplementary file 1 — Supplementary Tables. [file 41598_2021_95301_MOESM1_ESM.pdf]

Supplementary Material

refineR: A Novel Algorithm for Reference Interval Estimation from Real-World Data

Tatjana Ammer[1, 2*], André Schützenmeister[2], Hans-Ulrich Prokosch[1], Manfred Rauh[3], Christopher M Rank[2+] and Jakob Zierk[3, 4+]

[+]These authors contributed equally to the publication.

***Corresponding author:**
Tatjana Ammer, tatjana.ammer@roche.com

Affiliations:

[1]Chair of Medical Informatics, Friedrich-Alexander-University Erlangen-Nuremberg, Erlangen, Germany

[2]Roche Diagnostics GmbH, Penzberg, Germany

[3]Department of Pediatrics and Adolescent Medicine, University Hospital Erlangen, Erlangen, Germany

[4]Center of Medical Information and Communication Technology, University Hospital Erlangen, Erlangen, Germany

ALP (U/L)	Direct approach n = 120			Direct approach n = 400			kosmic n = 50,000; 100 seeds			refineR n = 50,000; 100 seeds		
Category	Overall [%]	Lower RI [%]	Upper RI [%]	Overall [%]	Lower RI [%]	Upper RI [%]	Overall [%]	Lower RI [%]	Upper RI [%]	Overall [%]	Lower RI [%]	Upper RI [%]
**<36; <115**	5.4	6.6	4.2	0.4	0.7	0.1	21.4	26.7	16.2	8.1	15.7	0.5
**36-38; 115-122**	16.5	15.3	17.6	8.3	9.3	7.3	16.1	18.9	13.3	8.2	12.5	4.0
**38-42; 122-136**	55.0	52.6	57.4	81.7	79.4	83.9	58.0	54.0	61.9	82.5	71.8	93.3
**42-44; 136-143**	15.6	17.6	13.6	9.1	10.0	8.1	3.3	0.4	6.2	1.0	0.0	1.9
**>44; >143**	7.6	7.9	7.2	0.6	0.6	0.6	1.2	0.0	2.4	0.2	0.0	0.3

Supplementary Table 1. Comparison of quality of results for simulated analyte "Alkaline phosphatase, ALP". The table shows for all considered methods (direct method with n = 120 and n = 400, *kosmic* and *refineR* algorithm) the distribution of RI estimates in the five evaluation categories (see Methods Table 1). The results are shown for the lower and upper reference interval as well as an average of both options for the overall amount. The percentages correspond to the color-coded matrix shown in Figure 3a. RI, reference interval.

CREA (µmol/L)	Direct approach n = 120			Direct approach n = 400			kosmic n = 25,000; 100 seeds			refineR n = 25,000; 100 seeds		
Category	Overall [%]	Lower RI [%]	Upper RI [%]	Overall [%]	Lower RI [%]	Upper RI [%]	Overall [%]	Lower RI [%]	Upper RI [%]	Overall [%]	Lower RI [%]	Upper RI [%]
**<42; <78**	2.8	4.2	1.3	0.1	0.2	0.0	13.1	23.3	2.8	9.9	19.4	0.5
**42-43; 78-81**	10.8	7.4	14.2	2.6	2.2	3.0	13.6	10.1	17.2	5.2	7.2	3.3
**43-47; 81-87**	72.4	75.3	69.6	94.3	95.6	93	69.5	66.4	72.5	81.2	73.4	89.1
**47-48; 87-90**	10.6	9.1	12	2.9	1.9	3.9	2.3	0.2	4.4	2.8	0.0	5.7
**>48; >90**	3.5	3.9	3.0	0.0	0.1	0.0	1.6	0	3.1	0.8	0.0	1.5

Supplementary Table 2. Comparison of quality of results for simulated analyte "Creatinine, CREA". The table shows for all considered methods (direct method with n = 120 and n = 400, *kosmic* and *refineR* algorithm) the distribution of RI estimates in the five evaluation categories (see Methods Table 1). The results are shown for the lower and upper reference interval as well as an average of both options for the overall amount. The percentages correspond to the color-coded matrix shown in Figure 3b. RI, reference interval.

Hb (g/dL)	Direct approach n = 120			Direct approach n = 400			kosmic n = 10,000; 100 seeds			refineR n = 10,000; 100 seeds		
Category	Overall [%]	Lower RI [%]	Upper RI [%]	Overall [%]	Lower RI [%]	Upper RI [%]	Overall [%]	Lower RI [%]	Upper RI [%]	Overall [%]	Lower RI [%]	Upper RI [%]
**<11.5; <15.4**	1.2	2.1	0.3	0.0	0.0	0.0	3.9	7.6	0.2	5.5	11.0	0.0
**11.5-11.8; 15.4-15.7**	13.3	17.2	9.5	4.2	7.5	0.8	10.5	16.9	4.2	12.3	24.6	0.0
**11.8-12.2; 15.7-16.3**	70.2	60.1	80.4	92.0	86.2	97.7	68.2	64.1	72.3	65.7	62.8	68.6
**12.2-12.5; 16.3-16.6**	14.3	19.7	8.9	3.8	6.3	1.4	13.2	10.9	15.4	11.4	1.5	21.4
**>12.5; >16.6**	1.0	1.0	0.9	0.0	0.0	0.0	4.2	0.4	7.9	5.0	0.0	10.0

Supplementary Table 3. Comparison of quality of results for simulated analyte "Hemoglobin, Hb". The table shows for all considered methods (direct method with n = 120 and n = 400, *kosmic* and *refineR* algorithm) the distribution of RI estimates in the five evaluation categories (see Methods Table 1). The results are shown for the lower and upper reference interval as well as an average of both options for the overall amount. The percentages correspond to the color-coded matrix shown in Figure 3c. RI, reference interval.

FT4 (pmol/L)	Direct approach n = 120			Direct approach n = 400			kosmic n = 50,000; 100 seeds			refineR n = 50,000; 100 seeds		
Category	Overall [%]	Lower RI [%]	Upper RI [%]	Overall [%]	Lower RI [%]	Upper RI [%]	Overall [%]	Lower RI [%]	Upper RI [%]	Overall [%]	Lower RI [%]	Upper RI [%]
**<10.8; <20**	0.4	0.9	0.0	0.0	0.0	0.0	8.1	16.1	0.0	10.3	20.7	0.0
**10.8-11.4; 20-21**	8.1	9.5	6.6	1.2	2.0	0.4	8.7	16.2	1.2	6.6	12.7	0.6
**11.4-12.6; 21-23**	81.9	78.3	85.5	97.7	96.6	98.8	76.3	66.9	85.7	78.9	66.6	91.3
**12.6-13.2; 23-24**	9.0	10.7	7.3	1.1	1.5	0.8	4.1	0.7	7.5	3.5	0.0	6.9
**>13.2; >24**	0.6	0.6	0.5	0.0	0.0	0.0	2.8	0.0	5.6	0.6	0.0	1.2

Supplementary Table 4. **Comparison of quality of results for simulated analyte "Free thyroxine, FT4".** The table shows for all considered methods (direct method with n = 120 and n = 400, *kosmic* and *refineR* algorithm) the distribution of RI estimates in the five evaluation categories (see Methods Table 1). The results are shown for the lower and upper reference interval as well as an average of both options for the overall amount. The percentages correspond to the color-coded matrix shown in Figure 3d. RI, reference interval.

TSH (µIU/mL)	Direct approach n = 120			Direct approach n = 400			kosmic n = 50,000; 100 seeds			refineR n = 50,000; 100 seeds		
Category	Overall [%]	Lower RI [%]	Upper RI [%]	Overall [%]	Lower RI [%]	Upper RI [%]	Overall [%]	Lower RI [%]	Upper RI [%]	Overall [%]	Lower RI [%]	Upper RI [%]
**<0.18; <2.9**	2.2	2.6	1.9	0.0	0.1	0.0	0.8	0.8	0.8	0.1	0.0	0.2
**0.18-0.22; 2.9-3.4**	16.2	18.1	14.3	6.2	8.9	3.5	3.8	4.9	2.8	0.2	0.0	0.4
**0.22-0.28; 3.4-4.6**	59.0	53.2	64.8	85.0	80.0	90.0	84.4	92.9	75.9	98.7	99.9	97.4
**0.28-0.32; 4.6-5.1**	15.8	20.0	11.5	8.4	10.8	6.0	4.5	1.4	7.5	1.0	0.0	1.9
**>0.32; >5.1**	6.8	6.1	7.4	0.4	0.3	0.6	6.5	0.0	13.0	0.0	0.0	0.1

Supplementary Table 5. Comparison of quality of results for simulated analyte "Thyroid-stimulating hormone, TSH". The table shows for all considered methods (direct method with n = 120 and n = 400, *kosmic* and *refineR* algorithm) the distribution of RI estimates in the five evaluation categories (see Methods Table 1). The results are shown for the lower and upper reference interval as well as an average of both options for the overall amount. The percentages correspond to the color-coded matrix shown in Figure 3e. RI, reference interval.

γ-GT (U/L)	Direct approach n = 120			Direct approach n = 400			kosmic n = 25,000; 100 seeds			refineR n = 25,000; 100 seeds		
Category	Overall [%]	Lower RI [%]	Upper RI [%]	Overall [%]	Lower RI [%]	Upper RI [%]	Overall [%]	Lower RI [%]	Upper RI [%]	Overall [%]	Lower RI [%]	Upper RI [%]
**<8; <41**	1.3	1.3	1.3	0	0	0	10.7	12.8	8.5	3.4	6.4	0.4
**8-9; 41-46**	14.9	11.5	18.3	4.5	3.2	5.8	13.8	9.2	18.5	3.3	5.1	1.6
**9-11; 46-54**	65.7	70.7	60.7	90	93.5	86.5	68.4	77.8	59.0	88.0	88.5	87.4
**11-12; 54-59**	14.9	14.6	15.2	5.4	3.3	7.5	4.2	0.2	8.2	4.5	0.0	9.1
**>12; >59**	3.3	1.9	4.7	0.1	0	0.2	3.0	0.0	5.9	0.8	0.0	1.5

Supplementary Table 6. Comparison of quality of results for simulated analyte "Gamma-glutamyltransferase, γ-GT". The table shows for all considered methods (direct method with n = 120 and n = 400, *kosmic* and *refineR* algorithm) the distribution of RI estimates in the five evaluation categories (see Methods Table 1). The results are shown for the lower and upper reference interval as well as an average of both options for the overall amount. The percentages correspond to the color-coded matrix shown in Figure 3f. RI, reference interval.

Biomarker	Sex	Age range [Years]	Nr. of samples	Unit	Instruments
ALP	Female	3-9	4,773	U/L	Roche cobas integra 800/c501
CREA	Female	15-18	5,571	µmol/L	Roche cobas integra 800/c501
Hb	Female	10-18	12,410	g/dL	SYSMEX XE-2100
FT4	Female	3-18	9,442	pmol/L	Roche cobas e411/e601
TSH	Male	3-18	9,744	µIU/mL	Roche cobas e411/e601
γ-GT	Female	12-18	6,228	U/L	Roche cobas integra 800/c501

Supplementary Table 7. Description of the pediatric patient datasets for the six analyzed biomarkers. The datasets were obtained from the laboratory information system of the Department of Pediatrics and Adolescent Medicine, University Hospital Erlangen, Erlangen, Germany.

Biomarker	Study/ Method	Age Range [y]	Nr. of samples	Lower RI			Upper RI			Reference
				Point Est	LCL	UCL	Point Est	LCL	UCL	
**ALP [U/L]**	CALIPER	1-10	200 (trans-ference study)	142	132	152	335	324	345	Estey et al., 2013
	Roche Package Insert	1-10	NA	142			335			Roche, 2019
	kosmic	3-9	4,773	115	115	155	299	287	356	
	refineR	3-9	4,773	127	112	135	345	315	357	
**CREA [enzymatic] [µmol/L]**	Austria*	14-17	39	51.3	50.4	52.2	87.5	86.6	88.4	Bogner et al., 2019
	CALIPER	15-20	48	39			92			Kulasingam et al., 2010
	CALIPER	15-19	200 (trans-ference study)	48	43	53	79	74	83	Higgins et al., 2016
	CALIPER	15-19	200 (trans-ference study)	41.3	37.6	45.0	71.0	67.3	74.7	Estey et al., 2013
	Roche Package Insert	>15	NA	45			84			Roche, 2020
	kosmic	15-18	5,571	40.1	38.7	43.9	73.6	70.6	76.3	
	refineR	15-18	5,571	40.7	39.7	44.0	76.5	74.0	77.3	
**Hb [g/dL]**	Austria*	14-17	39	9.00	8.84	9.16	14.60	14.44	14.76	Bogner et al., 2019
	Sysmex Package Insert	6-12	1,207	10.6			13.2			Hinzmann, R., Sysmex, 2010
		12-18	1,872	10.8			13.3			
	CALIPER*	4-14	247	11.2	10.9	11.3	14.1	14.0	14.6	Bohn et al., 2020
		14-21	99	11.2	10.7	11.5	15.1	14.8	15.4	
	kosmic	10-18	12,410	11.6	11.5	11.8	15.1	14.9	15.2	
	refineR	10-18	12,410	11.7	11.5	11.7	15.3	15.0	15.3	

**FT4 [pmol/L]**	CALIPER*	1-19	491	13	13	13	21	20	21	Bohn et al., 2019
	Roche LIFE Child	1-6	193	13.4	12.9	13.7	20.1	19.6	20.6	Roche, 2018
		6-11	243	12.9	12.4	13.1	19.7	19.3	20.4	
		11-20	206	11.6	11.2	12.0	19.6	19.3	20.8	
	CALIPER	0-5	208	10.90			36.30			Kulasingam et al., 2010
		5-15	238	10.43			27.07			
		15-20	36	9.99			18.73			
	kosmic	3-18	9,442	11.8	11.0	12.5	19.1	18.7	20.1	
	refineR	3-18	9,442	11.3	11.1	12.3	20.0	19.5	20.2	
**TSH [µIU/mL]**	CALIPER*	1-15	339	1.12	1.07	1.22	5.01	4.49	5.40	Bohn et al., 2019
		15-19	148	0.68	0.43	0.83	4.09	3.74	4.62	
	Roche LIFE Child	1-6	200	1.09	0.87	1.20	6.07	5.57	6.80	Roche, 2018
		6-11	250	1.13	1.04	1.24	5.34	4.95	5.82	
		11-20	208	1.01	0.97	1.12	5.09	4.58	5.39	
	CALIPER	0-5	189	0.84			6.22			Kulasingam et al., 2010
		5-10	47	1.18			5.33			
		10-15	118	0.76			4.20			
		15-20	26	0.64			5.37			
	kosmic	3-18	9,744	0.813	0.633	0.997	4.93	3.99	5.60	
	refineR	3-18	9,744	0.826	0.776	0.917	5.17	4.56	5.71	
**GGT [U/L]**	Austria*	14-17	39	5.00	4.43	5.57	27.00	26.43	27.57	Bogner et al., 2019
	CALIPER	1-19	200 (trans-ference study)	4	1	8	16	12	19	Higgins et al., 2016
	kosmic	12-18	6,228	8.02	7.11	8.44	20.7	18.6	21.6	
	refineR	12-18	6,228	8.37	8.0	8.60	21.5	19.6	22.5	

Supplementary Table 8. Comparison of reference intervals estimated with the *refineR* and *kosmic* algorithm to reference intervals published in literature. The table shows the different published or estimated reference intervals and the corresponding 95% (*90%) confidence intervals for the six analyzed biomarkers. Further, the age range in years (y) and the number of samples are shown as well as the reference to the corresponding study. RI, reference interval; Point Est, point estimate; LCL, lower confidence limit; UCL, upper confidence limit.
